# Epidemiology, Microbiological Diagnosis, and Clinical Outcomes in Pyogenic Vertebral Osteomyelitis: A 10-year Retrospective Cohort Study

**DOI:** 10.1093/ofid/ofy037

**Published:** 2018-03-10

**Authors:** Brian S W Chong, Christopher J Brereton, Alexander Gordon, Joshua S Davis

**Affiliations:** 1 Division of Medicine, John Hunter Hospital, New Lambton Heights, NSW, Australia; 2 School of Medicine and Public Health, University of Newcastle, NSW, Australia; 3 Global and Tropical Health Division, Menzies School of Health Research, Darwin, NT, Australia

**Keywords:** antibiotic treatment, diagnosis, discitis, vertebral osteomyelitis

## Abstract

**Background:**

Pyogenic vertebral osteomyelitis (PVO) is rising in incidence, but optimal methods of investigation and duration of antibiotic therapy remain controversial.

**Methods:**

We conducted a single-center retrospective cohort study of PVO at an Australian teaching hospital. We included all adults with a first episode of PVO between 2006 and 2015. PVO was defined based on the presence of prespecified clinical and radiological criteria. The main exposures of interest were investigation strategy and antibiotic treatment. The main outcome measures were duration of hospital admission, mortality during index admission, symptom resolution during index admission, and attributable readmission within 2 years.

**Results:**

Of 129 included patients, 101 (78%) had a causative organism identified. Patients with an identified pathogen were more likely to be febrile (75% compared with 29%, *P* < .001) and had a higher mean admission C-reactive protein (207 vs 54, *P* < .001) compared with patients without an identified pathogen. However, they were less likely to experience an adverse outcome (death or attributable readmission within 2 years; adjusted odds ratio, 0.36; 95% confidence interval, 0.13–0.99; *P* = .04). Open biopsy of vertebral tissue had a higher diagnostic yield (70%) than fine needle aspirate (41%) or core biopsy (30%). Despite receiving a median of 6 weeks of intravenous antibiotics, only 15% of patients had a full recovery on discharge from index admission.

**Conclusions:**

Clinical outcomes for patients with PVO were poor. Obtaining a microbiological diagnosis is associated with a better outcome. However, prospective and randomized studies are essential to establishing optimal investigation and treatment pathways.

Pyogenic vertebral osteomyelitis (PVO) presents significant diagnostic and therapeutic challenges, and its incidence appears to be gradually rising [1]. Clinical outcomes are frequently poor despite surgery and prolonged antibiotic treatment [2, 3]. The optimal duration of antibiotic treatment in patients with PVO remains controversial. Bernard et al. showed that 6 weeks of treatment was not inferior to 12 weeks of treatment [4]. Conversely, Park et al. subsequently found that a longer (≥8-week) course of antibiotic treatment reduced the rate of relapse in patients with certain high-risk characteristics [5]. Furthermore, the ideal diagnostic strategy is unclear.

Some patients do not have a pathogen identified despite extensive investigation and receive a prolonged course of broad-spectrum empirical antibiotic therapy, with implications for antimicrobial stewardship. Several previous studies have shown similar outcomes in patients who have culture-negative PVO compared with patients with microbiologically confirmed PVO [6, 7].

Despite the growing incidence of PVO and significant morbidity, there is a paucity of data from Australia. The goal of this study was to describe the current epidemiology, microbiology, and clinical outcomes of PVO in an Australian setting. We also aimed to explore the difference in outcomes for patients with directed vs empirical antimicrobial therapy.

## METHODS

### Study Design and Data Collection

We performed a retrospective, single-center observational study of PVO in a 650-bed Australian university teaching hospital. The study was approved by the local human research ethics committee (New South Wales Human Research Ethics Committee LNR approval number 15/12/16/5.06).

We identified patients with discharge coding for vertebral osteomyelitis, spondylodiscitis, disc infection, and epidural abscess (ICD-9-CM codes 324.1, 324.9, 730.28, 730.08, 730.2, 730.00, and 722.90–722.93) from January 2006 to December 2015. We then applied prespecified inclusion and exclusion criteria, which are listed later in this section. We reviewed the medical records and pathology results for all included patients. We also reviewed radiology reports from 14 days prior to index admission until discharge. We recorded all intravenous and oral antibiotics administered for more than 24 hours, from 7 days before the index admission until 2 years after.

Vertebral aspirates and biopsy samples are routinely processed by our clinical microbiology laboratory as follows: each specimen is Gram stained, then plated out onto blood, chocolate, MacConkey, and Columbia CNA solid agar, as well as thioglycolate broth. They are incubated in aerobic and anaerobic conditions for 5 days. Any bacteria that grow are identified using MALDI-TOF Bruker Biotyper (Bruker Daltonik, Germany) and Vitek 2 (bioMerieux, France).

Outcome measures were duration of index admission, mortality during index admission, symptom resolution upon discharge (we recorded the presence of fever, back pain, vertebral tenderness, limb weakness, sensory change, incontinence, and urinary retention), and attributable readmission within the next 2 years.

We collected data using purpose-built paper case report forms and collated data into a Microsoft Access (2016 version) database.

### Inclusion and Exclusion Criteria

Inclusion criteria were all of the following: (a) age 18 years or older; (b) radiological evidence of vertebral osteomyelitis on magnetic resonance imaging, computed tomography, or nuclear medicine scan as reported by a specialist radiologist; and (c) clinical evidence of vertebral osteomyelitis (2 or more of back pain, leg weakness, vertebral tenderness, and fever).

Exclusion criteria were any of: (a) contiguous osteomyelitis due to surgical site infection, wounds, or trauma; (b) index admission notes not available; (c) suspected or proven tuberculous vertebral osteomyelitis; (d) confirmation of alternative diagnosis to account for clinical and radiological features (eg, myeloma).

### Definitions

If a patient was admitted more than once, the first admission within any 2-year period was considered the index admission.

We determined a causative organism for each patient by reviewing blood and vertebral biopsy culture results. Organisms grown from other sites such as urine were disregarded. If these cultures were positive for more than 1 organism without an alternative explanation (eg, line sepsis), we recorded the patient as having a mixed infection. Organisms of low pathogenicity (eg, coagulase-negative *Staphylococci* and *Corynebacterium*) were disregarded unless they were isolated on more than 1 set of blood cultures or were the only organisms isolated on vertebral culture.

We defined an “adverse outcome” as either mortality during index admission or readmission attributable to PVO within 2 years. Immunosuppression was defined as the receipt of any of the following within the 3 months prior to admission: prednisone ≥0.5mg/kg/d for more than 14 days, cyclosporine, tacrolimus, sirolimus, azathioprine, mycophenolate, leflunomide, methotrexate, cyclophosphamide, monoclonal antibodies, cancer chemotherapy, or bone marrow transplant.

### Statistical Analysis

Normally distributed variables were compared using the Student *t* test, non-normal variables with the Mann-Whitney *U* test, and categorical variables were compared using chi-square tests. Logistic regression models were built with “adverse outcome” as the dependent variable and candidate covariates as the independent variables. Each variable was run in a univariate model, and those with a Wald *P* value of <.10 or that were judged to be clinically important were included in a multivariable model. We used Stata version 14 (Statacorp, College Station, TX) for statistical analysis. *P* values <.05 were considered significant.

## RESULTS

**Figure 1.**
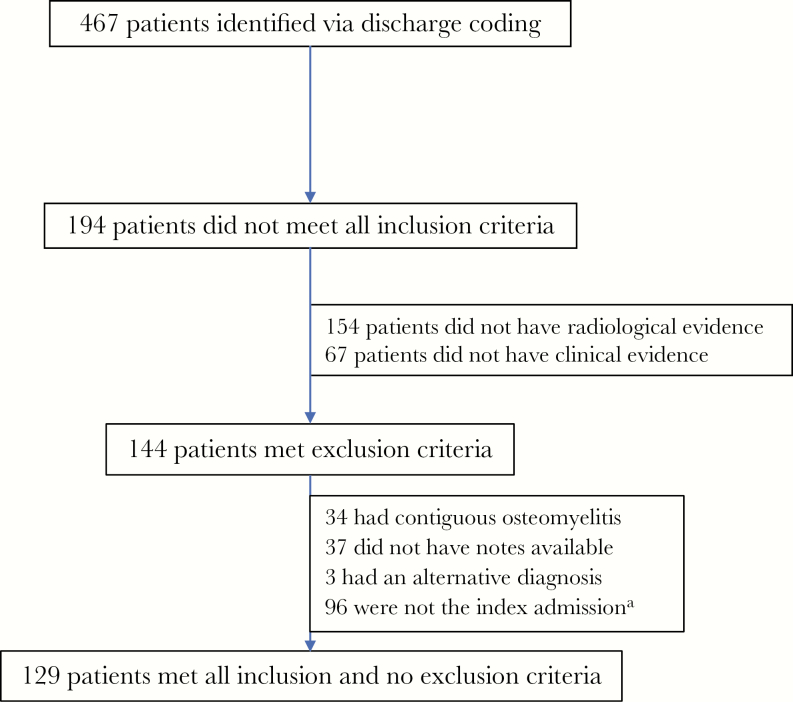
Recruitment flowchart. ^a^“Index admission” refers to the first admission, within a 2-year period, where the diagnosis of pyogenic vertebral osteomyelitis was established.

From January 2006 to December 2015, 467 patients were admitted with a compatible discharge diagnosis. Of these, we identified 129 patients for inclusion in the study (Figure 1). Eighty-eight (68%) patients were male, and the mean age (range) was 61 (18–92) years; 101 (78%) patients had an organism identified, and 28 did not. The 2 groups were well matched with regards to baseline demographics, apart from the presence of iatrogenic immunosuppression (Table 1). With regards to clinical features on presentation and initial investigations, patients who had an organism identified were much more likely to be febrile (75% vs 29%, *P* < .001) and had a higher C-reactive protein on admission (207 vs 54, *P* < .001), but the 2 groups were otherwise similar. Of the 28 patients with no causative organism identified, histology was only available for 5; of these, 3 showed chronic inflammation, 1 showed acute inflammation, and 1 was nondiagnostic.

**Table 1.** Demographics, Comorbidities, Presenting Features, Management, and Outcomes of Pyogenic Vertebral Osteomyelitis Among 129 Adult Patients Admitted to a Teaching Hospital in Australia from 2006 to 2015, Divided According to Whether an Organism was Identified

	No Organism Identified (n = 28)	Organism Identified (n = 101)	*P*
Demographics and comorbidities			
Male sex, No. (%)	18 (64)	70 (69)	NS
Age, mean (SD), y	58 (17)	62 (15)	NS
Diabetes, No. (%)	7 (25)	24 (24)	NS
Chronic renal disease, No. (%)	0 (0)	7 (7)	NS
Intravenous drug user, No. (%)	4 (14)	9 (9)	NS
Chronic liver disease, No. (%)	0 (0)	4 (4)	NS
Immunosuppression, No. (%)	3 (11)	2 (2)	.03
Malignancy, No. (%)	1 (4)	4 (4)	NS
Clinical features at presentation, No. (%)			
Fever	8 (29)	76 (75)	<.001
Back pain	27 (96)	101 (100)	NS
Vertebral tenderness	16 (57)	41 (41)	NS
Limb weakness	8 (29)	36 (36)	NS
Urinary retention	2 (7)	11 (11)	NS
Investigations			
Admission C-reactive protein, median (IQR), mg/dL	54 (19–108)	207 (120–272)	<.001
Vertebral samples taken, No. (%)	15 (54)	42 (42)	NS
Blood cultures taken, No. (%)	25 (89)	99 (99)	NS
Treatment			
Antibiotics commenced prior to admission, No. (%)	2 (7)	10 (10)	NS
Duration of IV antibiotics, median (IQR), d	42 (39–47)	43 (42–49)	NS
Duration of PO antibiotics, median (IQR), d	42 (37–100)	42 (35–84)	NS
Surgical intervention, No. (%)	7 (25)	40 (40)	NS
Outcomes			
Hospital mortality, No. (%)	1 (4)	4 (4)	NS
1-y mortality, No. (%)	1 (4)	10 (10)	NS
Length of index admission, median (IQR), d	44 (25–50)	43 (27–51)	NS
Attributable readmission within 2 y, No. (%)	8 (28)	15 (15)	.11
C-reactive protein, median (IQR) at discharge, mg/dL	12 (6–31)	23 (9–53)	NS
Complete recovery at discharge, No. (%)	4 (14)	15 (15)	NS
Back pain at discharge, No. (%)	22 (79)	79 (78)	NS
Vertebral tenderness at discharge, No. (%)	0 (0)	5 (5)	NS
Limb weakness at discharge, No. (%)	3 (11)	26 (26)	NS
Incontinence at discharge, No. (%)	2 (7)	6 (6)	NS

For statistical analysis, normally distributed variables were compared using the Student *t* test, non-normal variables with the Mann-Whitney *U* test, and categorical variables with the chi-square test.

Abbreviations: IQR, interquartile range; IV, intravenous; PO, per os.

One hundred twenty-four (96%) patients had blood cultures collected at some point between 14 days prior to index admission and discharge. Blood cultures were positive in 83 (67%) of these patients. Vertebral samples were collected for 57 (44%) patients. Open biopsy had a significantly higher yield than either FNA or core (Table 3). 16s RNA polymerase chain reaction (PCR) on vertebral samples was nondiagnostic in all 5 patients in whom it was done.

Of the 101 patients with an identified organism, methicillin-sensitive *Staphylococcus aureus* was the most prevalent (Table 2), and 88 (87%) patients had a Gram-positive organism.

**Table 2.** Details of Pathogens Identified (n = 101)

Pathogen	No.^^a^^
Gram-positive	88
*Staphylococcus aureus*	
Methicillin-sensitive	63
Methicillin-resistant	3
Coagulase-negative *Staphylococcus*	2
Viridans group *Streptococcus*	5
Beta-hemolytic *Streptococcus*	4
Milleri group *Streptococcus*	4
*Streptococcus pneumoniae*	2
*Enterococcus* sp.	2
*Bacillus* sp.	1
*Peptostreptococcus* sp.	1
*Propionibacterium* sp.	1
Gram-negative	7
*Escherichia* sp.	3
*Burkholderia* sp.	1
*Fusobacterium* sp.	1
*Klebsiella* sp.	1
*Serratia* sp.	1
More than 1 organism identified^**^b^**^	6

^a^As n = 101, No. is roughly equal to the percentage of each organism.

^b^Pathogens identified are as follows: patient 1 – Beta-hemolytic *Streptococcus* + milleri group *Streptococcus*; patient 2 – *Escherichia coli* + milleri group *Streptococcus*; patient 3 – *Corynebacterium* sp. + coagulase-negative *Staphylococcus*; patient 4 – methicillin-sensitive *Staphylococcus aureus* + beta-hemolytic *Streptococcus*; patient 5 – *Klebsiella* sp. + gram-positive cocci (unidentified); patient 6 – *Escherichia* sp. + *Bacteroides* sp. + *Pseudomonas* sp.

**Table 3.** Diagnostic Yield of Different Vertebral Biopsy Methods^a^

Method	Diagnostic Yield (%)
Open biopsy	21 of 30 (70)
Fine needle aspirate	7 of 17 (41)
Core biopsy	3 of 10 (30)

*P* values: open vs FNAB *P* = .05; open vs core *P* = .02; FNAB vs core *P* = .56.

^a^Not mutually exclusive.

Overall outcomes were poor regardless of whether an organism was identified (Table 1). Despite receiving a median of 43 days of intravenous antibiotics, only 15% of patients had complete recovery on discharge, and 78% of patients had ongoing back pain. Those with no organism identified had a nonsignificant trend to an increased risk of attributable readmission (28% vs 15%, *P* = .11).

On univariate analysis (Table 4), the predictors of an adverse outcome were limb weakness on presentation (odds ratio [OR], 2.9; 95% confidence interval [CI], 1.21–6.76; *P* < .05) and requiring a therapeutic intervention (OR, 2.66; 95% CI, 1.27–7.11; *P* < .05). Therapeutic interventions included drainage of epidural or paraspinal abscesses, laminectomy, vertebrectomy, and spinal fusion. On multivariate analysis, patients requiring a therapeutic intervention were more likely to have an adverse outcome (adjusted OR [aOR], 2.78; 95% CI, 0.01–7.01; *P* = .04), whereas having a pathogen identified was associated with a better outcome (aOR, 0.36; 95% CI, 0.13–0.99; *P* = .04).

**Table 4.** Predictors of an Adverse Outcome^a^ on Univariate and Multivariate Analysis

Variable	OR (95% CI)	aOR (95% CI)
Male **sex**	0.56 (0.23–1.31)	
Age, per y of age	1.005 (0.977–1.032)	
Diabetes	1.08 (0.41–2.86)	
Chronic renal disease	1.49 (0.27–8.13)	
IVDU	0.64 (0.13–3.05)	
Immunosuppression	0.91 (0.10–8.45)	
Limb weakness at presentation	2.9 (1.21–6.76), *P* < .05	2.2 (0.84–5.65), *P* = .10
Sensory loss at presentation	0.84 (0.29–2.47)	
Urinary incontinence	2.53 (0.76–8.44)	
Urinary retention	0.58 (0.12–2.74)	
Required therapeutic intervention	2.66 (1.27–7.11), *P* < .05	2.78 (1.01–7.01), *P* = .04
Back pain on discharge	0.61 (0.11–3.37)	
Causative organism identified	0.48 (0.19–1.24)	0.36 (0.13–0.99), *P* = .04
IV antibiotic duration, per d	0.99 (0.96–1.02)	
IV antibiotic duration >4 w**k**	0.62 (0.20–1.94)	
Baseline C-reactive protein, per mg/dL	1.001 (0.997–1.004)	
Discharge C-reactive protein, per mg/dL	1.003 (0.994–1.012)	
*Staphylococcus aureus* as causative organism	0.62 (0.26–1.43)	

Abbreviations: aOR, adjusted odds ratio; CI, confidence interval; IV, intravenous; IVDU, intravenous drug user; OR, odds ratio.

^a^Adverse outcome was defined as mortality during index admission or attributable readmission within 2 years.

## DISCUSSION

### Main Findings

In this retrospective, single-center study of PVO, we found a predominance of Gram-positive pathogens (mainly methicillin-sensitive *Staphylococcus aureus*), prolonged antibiotic treatment, and poor clinical outcomes regardless of whether a pathogen was identified. Furthermore, patients who did not have a pathogen identified were at increased risk of an adverse outcome.

### Comparison with Literature

To the authors’ knowledge, this cohort study is the largest of its kind in an Australian setting, and 6 times larger than the only other Australian study on vertebral osteomyelitis [8]. In addition, our cohort size and demographics are similar to current international publications [3, 5, 9–13].

In keeping with the literature, the majority of our patients were elderly males [14]. Back pain was a nearly universal presenting feature [14], and blood cultures were positive in roughly two-thirds of cases [5, 15]. Methicillin-sensitive *S. aureus* was the most common pathogen [15, 16]. Compared with previous studies, we found a lower rate of methicillin-resistant *S. aureus* in our cohort [5, 9, 10]. Our patients received a median of 6 weeks of intravenous antibiotics, followed by 6 weeks of oral antibiotics. Bernard et al. reported in 2015 on the noninferiority of a 6-week antibiotic regimen compared with a 12-week regimen [4]. In their study, patients received a median of 2 weeks of intravenous antibiotics. It is worth noting that French antibiotic guidelines advocate for the highly bioavailable combination of rifampicin and fluoroquinolones when practical, whereas Australian guidelines do not [17].

### Implications of Findings

Patients who ended up having an organism identified were more likely to be febrile (75% vs 29%, *P* < .001) and had a higher C-reactive protein count on presentation (207 vs 54, *P* < .001). There are several possible explanations for this finding. The first is that patients who did not have an organism identified had low-grade infection, or infection with fastidious organisms that were not identified on routine culture. 16s RNA PCR was not utilized to a significant extent in this subgroup, but it is more likely to be requested for current and future cases. In our cohort, none of the 5 samples that did have 16s RNA PCR requested were positive. The second possibility is that patients who did not have an organism identified did not, in fact, have vertebral osteomyelitis despite meeting clinical and radiological criteria. They could have had noninfectious etiologies such as malignancy, reactive osteitis, or stress injuries. This highlights the difficulty in making a definite diagnosis in some cases. Prior use of antibiotics is unlikely to explain the lack of a microbiological diagnosis in some patients as only 7% of these patients had received antibiotics in the 7 days prior to hospital admission, compared with 10% in the group where a causative organism was identified.

With regards to the various vertebral sampling methods, open biopsy had the highest yield (70% compared with 41% for fine needle aspirate and 30% for core biopsy). This is likely explained by the larger sample obtained by an open method compared with a radiology-guided biopsy, but also could be due to the fact that open biopsy samples were usually collected during a therapeutic intervention, and these patients were more likely to have a severe bacterial infection.

Vertebral osteomyelitis is a costly and morbid disease. Regardless of whether there was an organism identified, patients received a median of 42 days of intravenous antibiotics, either in the hospital or via the community antibiotic infusion program. The median duration of hospital admission was 43 days, and only 15% of patients were completely cured at the time of discharge. Having an organism identified did appear to be protective. There are several possible explanations for this finding. First, patients with a microbiological diagnosis may have benefited from targeted antibiotic therapy, and this may have led to a better outcome. Another possibility is that patients without a microbiological diagnosis had an indolent course prior to admission. This is supported by our finding that patients without a microbiological diagnosis were less likely to be febrile and had lower mean C-reactive proteins on admission. Patients with a chronic infection would, by definition, have a more protracted recovery course and therefore have poorer outcomes according to our study definition. Finally, patients without a microbiological diagnosis may not have had an infection, and therefore would not have improved with antibiotic treatment.

Our results differ to those of earlier studies, which found that patients with culture-negative PVO had equal or better outcomes compared with those with microbiologically confirmed PVO [6, 7]. One possible explanation for this disparity is differing definitions for failure between studies. Earlier research defined treatment failure as death, clinical relapse, or microbiological relapse, whereas we defined an adverse outcome as either death or attributable readmission within 2 years. This definition was intentionally broad and thus aimed to include patients who did not have definite relapse but required readmission for pain management, persistent disability, or repeat investigation, encompassing a more realistic representation of the overall impact of PVO to the patient and the health system.

Current Australian [17] and American [18] guidelines recommend intravenous vancomycin and a third- or fourth-generation cephalosporin for the empirical treatment of vertebral osteomyelitis. In our institution, flucloxacillin would provide adequate cover for 78% of patients who ultimately ended up having a pathogen identified. This has an implication for future antibiotic guidelines and antimicrobial stewardship, as we may be able to avoid unnecessarily broad-spectrum empirical treatment.

### Strengths and Limitations

Our study describes the largest cohort of vertebral osteomyelitis cases in Australia [8]. A high proportion of patients had complete data available, as we have excluded patients with insufficient records from our study.

This study is limited by its observational and retrospective design and thus can’t account for potential unmeasured confounders. In addition, this is a single-center study, and hence both local microbial patterns and clinical practice may vary in other regions. Importantly, however, both local microbial epidemiology and practice are consistent with national and international evidence [17] and therefore reflective of standard practice in Australia.

### Conclusions and Future Directions

Vertebral osteomyelitis is a common and potentially devastating infection with a significant burden of attributable morbidity [10], but there is little high-level evidence to guide investigation and management. Our findings highlight the importance of making every possible effort to identify a causative organism and avoiding empirical treatment if possible. More evidence is needed to clarify the optimal duration of antibiotic treatment. This study highlights the significant morbidity for patients with vertebral osteomyelitis in Australia and adds further weight to the importance of obtaining a microbiological diagnosis with which to guide treatment [4, 5]. Prospective and randomised trials are needed to strengthen the evidence base for this common infection.
